# Hierarchical Doped Gelatin-Derived Carbon Aerogels: Three Levels of Porosity for Advanced Supercapacitors

**DOI:** 10.3390/nano10061178

**Published:** 2020-06-17

**Authors:** Ayshuwarya Kandasamy, Tamilselvi Ramasamy, Ayesha Samrin, Padmanathan Narayanasamy, Ramesh Mohan, Olha Bazaka, Igor Levchenko, Kateryna Bazaka, Mandhakini Mohandas

**Affiliations:** 1 Center for Nanoscience and Technology, Anna University, Chennai 600025, India; ayshu92@gmail.com (A.K.); tamil.anjac@gmail.com (T.R.); ayesha.samu@gmail.com (A.S.); 2 Department of Physics, Karpagam Academy of Higher Education, Coimbatore 641021, India; padmanmsc@gmail.com; 3 Smart Sensors, CSIR-Central Electronics Engineering Research Institute, Pilani, Rajasthan 333031, India; ramesh_pondi@yahoo.co.in; 4 School of Science, RMIT University, PO Box 2476, Melbourne, VIC 3001, Australia; olga.bazaka@jcu.edu.au; 5 Plasma Sources and Application Centre/Space Propulsion Centre Singapore, NIE, Nanyang Technological University, Singapore 637616, Singapore; levchenko.igor@nie.edu.sg; 6 Science and Engineering Faculty, Queensland University of Technology, Brisbane, QLD 4000, Australia; 7 Research School of Electrical, Energy and Materials Engineering, The Australian National University, Canberra, ACT 2601, Australia

**Keywords:** supercapacitors, graphene based aerogel, porosity

## Abstract

Nitrogen-doped graphene-based aerogels with three levels of hierarchically organized pores were prepared via a simple environmentally friendly process, and successfully tested in supercapacitor applications. Mesopores and macropores were formed during the aerogel preparation followed by carbonization and its chemical activation by potassium hydroxide (KOH). These mesopores and macropores consist of amorphous carbon and a 3D graphene framework. Thermal treatment at 700 °C, 800 °C, 900 °C in N_2_ atmosphere was done to etch out the amorphous carbon and obtain a stable N-doped 3D graphene. Specific capacitance values obtained from the electrochemical measurements are in the range of 232–170 F× g^−1^. The thus fabricated structures showed excellent cyclic stability, suggesting that these materials have potential as electrodes for solid asymmetric supercapacitors.

## 1. Introduction

Hierarchical nanomaterials have attracted notable attention for their potential in the fields of high-performance supercapacitors [1,2], renewable energy generation and storage devices [3,4], memory storage devices, space electric propulsion systems [5,6] and many other applications [7,8]. In energy storage applications, complex hierarchical nanomaterials have been used as supercapacitor materials due to a favorable combination of high-power density, extended cycle life and a suitable safety profile. For supercapacitors, porous carbons provide an attractive alternative to transition metal oxides and conducting polymers as an electrode material [9,10,11]. While transition metal oxide and conducting polymers have high-energy density and supercapacitance, they also exhibit low stability. In contrast, porous carbons have a high surface area and a well-developed microstructure which affords good supercapacitance [12,13,14,15], as well as the high stability characteristic of this family of materials [16,17,18,19].

Driven by the growing interest in the green low-cost synthesis of advanced materials from minimally processed resources, recent times have seen the synthesis of porous carbon intended for supercapacitor and other electrode applications from a wide range of bio resources, from cellulose and starch, to chitosan, oils, alginate, silk, and collagen, to name a few [20,21,22,23,24]. Gelatin is a widely available biopolymer which can be used to form micro-, meso- and macro-hierarchical porous structures for the subsequent synthesis of porous carbons [25,26,27]. This is because the porosity of gelatin can be maintained due to its thermal stability. The highly porous structure of a gelatin-derived framework also allows for metal ions to penetrate and dope the carbon structure, thereby further enhancing its efficiency as electrode material. Junke Ou et al. developed hierarchical porous carbon materials (HPCMs) with nitrogen doping for sodium ion battery applications from the gelatin by potassium hydroxide (KOH) activation [28,29,30,31]. Inspired by this body of work, in this study, a novel 3D nitrogen self-doped graphene-based carbon aerogel is synthesized directly from gelatin (as a low-cost abundant carbon source) by first cross-linking it with glutaraldehyde, followed by deep freezing, carbonization and activation. Thermal treatment of dry-frozen aerogels at 700, 800 and 900 °C in N_2_ atmosphere is used to attain a complex structure that features a combination of a graphene framework with amorphous carbon [32]. The KOH activation is performed to remove the amorphous carbon, leaving behind a 3D nitrogen self-doped graphene.

## 2. Materials and Methods

**Figure 1:**
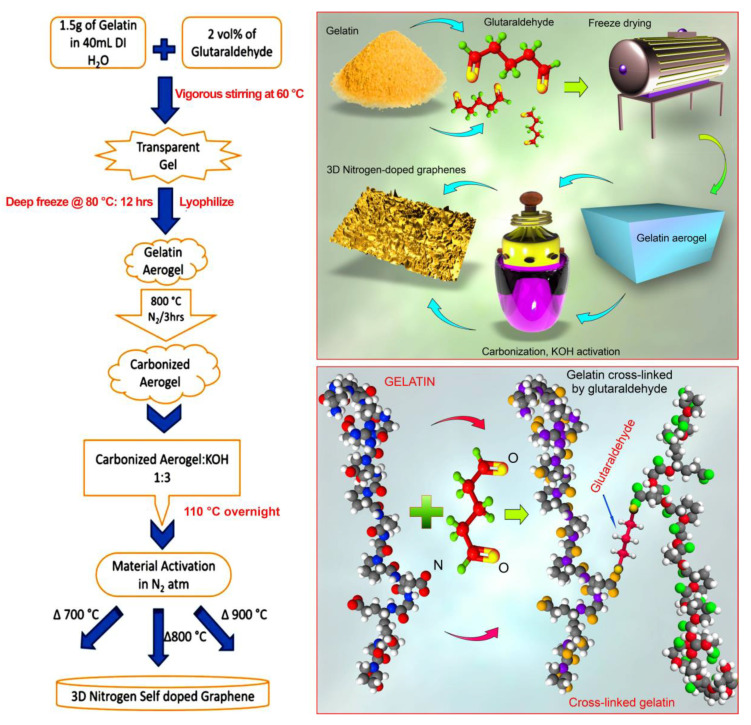
Step-by-step synthesis of 3D nitrogen self-doped graphene-based carbon aerogels involves the cross linking of gelatin and glutaraldehyde, followed by the deep freezing of the gel (shown in the bottom right image). Top right image shows an overall experimental approach.

The materials required for the synthesis of 3D graphene carbon aerogels, namely gelatin, glutaraldehyde (CHDS(CH_2_CHO)_2_) and potassium hydroxide (KOH), were procured from Sisco Research Laboratories Pvt. Ltd. (Mumbai, India). Gelatin powder derived from bovine skin was used without further purification. This form of gelatin is produced by means of the alkaline digestion of collagen, and is a heterogeneous mixture of collagen-derived proteins of variable molecular weight rich in proline, glycine, and hydroxyproline. The 3D nitrogen self-doped graphenes were synthesized following the steps outlined in Figure 1 [26,33]. In each experiment, for every 1.5 g of raw gelatin dissolved in 40 mL of water used, approximately 1.06 g of aerogel was synthesized. The mass yield of the final carbon material from the gelatin was approximately 70.6%, with the carbon yield of approximately 42.8% (based on the estimated elemental composition of gelatin of ~67% C, 11.5% Ni, and 21.5% O). It should be noted that since gelatin is a natural product used without further processing, both the nitrogen and water content of gelatin can vary significantly (due to method of extraction, type of starting material, age and breed of animals, etc.), affecting the values of both the mass and the carbon yield. In general, gelatin represents a heterogeneous mixture of protein >90%, lipids <1%, ash ~1%, and water. The moisture content of 10–13% and even up to 15% was often observed (with a possibility that the latter may change rapidly with extremes in ambient conditions, particularly humidity, due to the highly hygroscopic nature of gelatin). The protein fraction itself is a mixture of high average molecular weight (ca. 50,000 to 80,000) proteins, with an average nitrogen content of 16%, and glycine, proline, hydroxyproline and alanine accounting for >66% of all amino acids.

The the synthesized 3D self-doped graphene structures were characterized by X-ray diffraction (XRD) analysis by means of a Rigaku Miniflex II-c X-ray diffractometer (Rigaku Denki, Ltd., Tokyo, Japan) with Cu K radiation of wavelength 0.154 nm. The powders of thus synthesized 3D graphene aerogels were loaded into the device and scanned through a range of 2θ angles from 10° to 80°. The XRD data were used to estimate the average crystal size (*d*_XRD_) using the Scherrer equation.

Scanning Electron Microscopy (SEM, VEGA3 TESCAN, Tescan Co. Ltd., Brno, Czech Republic) was used to obtain the details of the surface morphology. Fourier transform infrared (FTIR) spectroscopy was performed using a Shimadzu IRPrestige-21 infrared spectrometer over the range of 4000–500 cm^−1^. Brunauer–Emmett–Teller (BET, Micromeritics ASAP 2030, Norcross, GA, USA) specific surface area was estimated using the N_2_ adsorption/desorption isotherm. The porosity of the thus produced porous carbon aerogels were estimated by N_2_ adsorption/desorption experiments conducted at the temperature of −196 °C using the Accelerated Surface Area & Porosimetry System (Micromeritics ASAP 2030, Norcross, GA, USA). Prior to these studies, the samples were outgassed in a vacuum at 398 K for 24 h. The total amount of N_2_ adsorbed by the graphene-based aerogel was assumed to correspond to the approximate total volume of the micro- and meso-pores. The total amount of adsorbed N_2_ was calculated at a relative pressure of P/P_0_ = 0.99. The Barrett–Joyner–Halenda (BJH) model was used to calculate the pore size distribution within the 3D framework. The nitrogen isotherms were used to determine the pore size distributions in the meso- (2–50 nm) and micropore (<2 nm) regimes as a function of the temperature treatment following the approach previously reported for similar graphene-based and other carbon aerogels [34,35,36,37]. The electrochemical performance of the synthesized electrode materials was characterized by cyclic voltammetry (CV), galvanostatic charge/discharge and electrochemical impedance spectroscopy (EIS). These studies were performed by means of Autolab PGSTAT302 (Eco-Chemie, Utrecht, The Netherlands).

To prepare the electrodes, a slurry was made by mixing 5 mg of the thus synthesized material and 0.5 mg of poly-vinylidenefluoride (PVDF). Following 15 min of grinding, the mixture was added to 0.1 mL of N-methyl pyrrolidinone (NMP) solution, and then subjected to 20 min of sonication [38]. The surface of nickel foil was roughened using sand paper to eliminate the oxide present on its surface. Then, the paste-like sonicated mixture was coated onto the foil and dried in the oven at 120 °C for 12 h. The working electrode was then ready for calibration.

The electrochemical behavior of the thus prepared electrode materials was characterized by cyclic voltammetry (CV), galvanostatic charge/discharge and electrochemical impedance spectroscopy (EIS). To do this, Autolab PGSTAT302 (Eco-Chemie, Utrecht, The Netherlands) was operated in a three-electrode mode, with the potential ranging from −0.6 to 0.6 V. For the counter electrode, a platinum foil was used, whereas a saturated calomel reference electrode (SCE) was used as a reference electrode. EIS data were collected over 0−25 Hz frequency at an open circuit potential. The N-doped 3D-graphene aerogel on Ni foil played the role of the working electrode. The specific capacitance (*C*_sp_) values were estimated from the following equation:

*C*_sp_ = ∫ *I*d*t*/*mν* (1)

*C*_sp_ = *It*/*mV* (2)

where *I* (A) represents the current used for the charge/discharge, *t* (s) is the time of the discharge, *m* (g) is the weight of the working electrode, and *ν* corresponds to the scan rate.

## 3. Results and Discussion

### 3.1. Morphological Analysis

**Figure 2:**
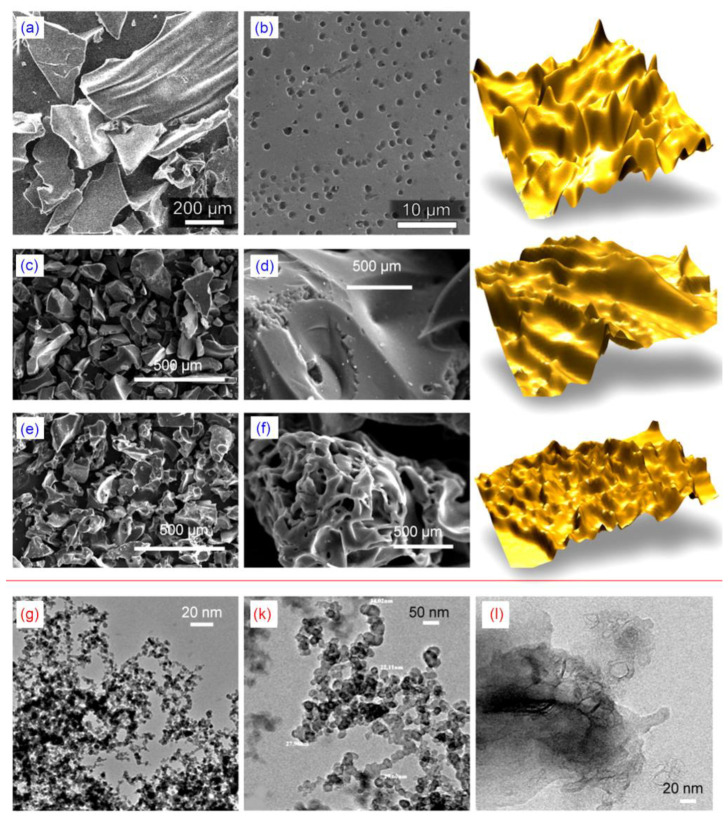
Upper panel: SEM images and 3D reconstructions of nitrogen self-doped graphene-based carbon aerogels activated at (**a**,**b**) 700 °C, (**c**,**d**) 800 °C, and (**e**,**f**) 900 °C. Lower panel (**g**,**k**,**I**): TEM images of the 3D nitrogen self-doped graphene-based carbon aerogel activated at 800 °C. The 3D reconstructions were made by translating the grayscale images into 3D using the Gwyddion^©^ visualization and analysis free software (released by GPL license), to illustrate the morphology only, with arbitrary height.

To study the morphology in detail, SEM images were collected on samples activated at different temperatures (of 700, 800 and 900 °C). It is evident from Figure 2a–f, that the porosity of the material increased substantially with an increase in the activation temperature. Figure 2f also shows a significant increase in the pore size and roughness due to higher etch rates of KOH at 900 °C promoted by the high reaction between the etchant and the carbon source. It should also be noted that prior to carbonization, the aerogels produced from the gelatin had a characteristic porous structure rich in 3-dimensional interconnected channels that were relatively uniform with respect to their size and distribution. The well resolved 3D interconnectivity of the pores is clearly observed in Figure 2f which corresponds to the aerogels activated at 900 °C. The carbonization step forces the breakdown of these uniform 3D interconnected channels and their subsequent re-assembly into a porous scaffold characterized by a distinct morphology, where the majority of channel walls undergo a transformation into flat nanosheets. These nanosheets are arranged into stacks and have a certain orientation, producing a 3D network with nanoscale features, as seen in Figure 2.

Figure 2 (lower panel, g, k, and I) shows the high resolution transmission electron microscopy (HRTEM) images taken on 3D nitrogen self-doped graphene-based carbon aerogel samples activated at 800 °C. TEM images for 800 °C were taken because this material has a well-developed 3D network structure, as shown in the SEM images magnified at 20 nm. The SEM and HRTEM images depicted in Figure 2 showed a sheet-like morphology which was transparent under electron beam irradiation, suggesting that the thus fabricated nanosheets were thin.

### 3.2. Brunauer−Emmett−Teller (BET) and Barrett−Joyner−Halenda (BJH) Analyses

**Figure 3:**
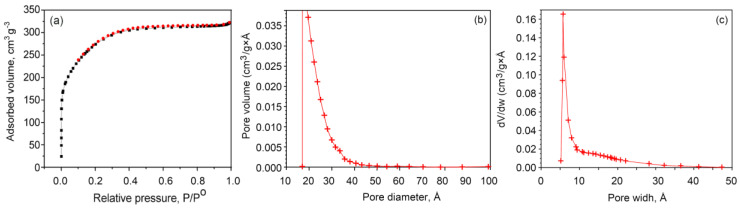
(**a**) N_2_ adsorption and desorption, and (**b**,**c**) the pore-size distribution of the 3D nitrogen self-doped graphene-based carbon aerogel activated at 800 °C: (**b**) Barrett−Joyner−Halenda pore distribution curve of the pore volume as a function of the pore diameter. (**c**) Horvath−Kawazoe differential pore volume plot.

A typical N_2_ adsorption/desorption isotherm for the carbon aerogel is presented in Figure 3a; it represents a type IV isotherm. From the plot, it is evident that the major adsorption takes place at a low relative pressure (<0.2), with the higher relative pressure region of the plot being virtually a horizontal plateau. These results suggest a carbon structure characterized by relatively high microporosity, with a narrowly distributed pore size, with a limited amount of mesopores present as indicated by a hysteresis loop that occurs at relative pressures of 0.4–0.9.

It should be noted that the shape of the hysteresis loop is narrow, suggesting minimal pore blocking within the aerogel network (since the latter would generally result in a significantly wider hysteresis loop). For materials with a narrow hysteresis loop, the modeling of either the adsorption and desorption branches of nitrogen isotherms will produce similar pore size distributions, whereas the latter results will be notably different for materials with a prominent hysteresis loop. Figure 3b shows the representative pore distribution for the aerogels where the curve is the result of the modeling of the desorption isotherm using the Cohan equations following the Barrett−Joyner−Halenda method, an approach previously used to model the pore distribution for similar graphene-based and other carbon aerogels [34,35,36,37].

The differential pore-volume distribution patterns for the micro, meso and macropore regions were estimated using the Horvath−Kawazoe model, a method frequently used to determine pore-size distribution in the micropore range [39], by employing the built-in software in the Accelerated Surface Area & Porosimetry System. The approach was based on the thermodynamics of adsorption, where the mean free energy change of adsorption that that takes place at the instance of the adsorbate molecule, being transferred from the gas to the solid in the pore, is used to estimate the analytical pore-filling correlation [40]. The graph in Figure 3c of the pore-volume distribution vs. pore diameter is a function, where, for any given pore diameter range, the area under the function reflects the total volume of pores over that range [40].

When activated at 700 °C, the 3D graphene nitrogen self-doped carbon aerogels had specific surface areas of 230 m^2^/g, total pore volumes of 0.24 cm^3^/g and the average pore diameter of 4.55 nm (Table 1). When activated at 800 °C, the aerogels had specific surface areas of 1539 m^2^/g, total pore volume of 0.88 cm^3^/g, and the average pore diameter of 7.5–15 nm, as shown in Figure 3. When activated at 900 °C, the material had a specific surface area of 1420 m^2^/g, total pore volumes of 0.78 cm^3^/g, and the average pore diameter of 17 nm. Hence, when activated at 800 °C, the aerogel had a greater specific area than that activated at either 700 °C and 900 °C. It should be noted that the choice of appropriate characterization techniques and testing parameters is critical in obtaining data that accurately account for the behavior of these complex structures [41].

Increasing the specific area of the porous material can notably improve the specific capacitance of the material. The average pore diameter of the material shows the presence of some mesopores when it is activated at 800 °C, which improves the specific capacitance. The increase in the surface area was due to the activation of KOH because the etching rate of KOH on the carbon-based aerogel is greater, and it etches all the amorphous carbon leaving the pores in the sheet. Hence, the pore diameter is increased to 19.3 nm when compared to that of the material activated at 700 °C. This increase in the surface area is one of the main factors for the observed increase in the supercapacitance, therefore this material (activated at 800 °C) was selected for all cyclic voltammetry studies.

### 3.3. Structural and Chemical Analysis

**Figure 4:**
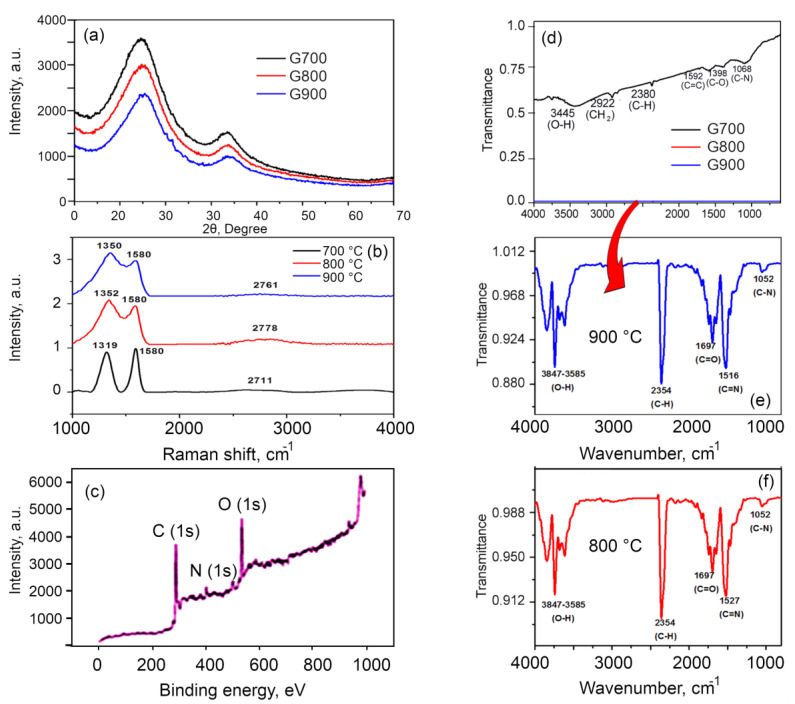
Characterization of the nitrogen self-doped graphene-based carbon aerogels. (**a**) XRD patterns of carbon aerogel activated at 700, 800, and 900 °C. (**b**) Raman spectroscopy analysis of the 3D nitrogen self-doped graphene-based carbon aerogels activated at 700, 800, and 900 °C. (**c**) XPS spectroscopy analysis of the 3D nitrogen self-doped graphene-based carbon aerogel activated at 800 °C. (**d**) Fourier transform infrared (FTIR) spectra of the 3D graphene nitrogen self-doped carbon aerogels activated at 700, 800, and 900 °C; (**e**,**f**) Enlarged FTIR spectra of the 3D graphene nitrogen self-doped carbon aerogels activated at 800 and 900 °C.

Figure 4a shows the XRD patterns of the 3D nitrogen self-doped graphene-based carbon aerogels activated at 700 °C, 800 °C and 900 °C. When activated at 700 °C, the 3D nitrogen self-doped carbon aerogel showed a broad diffraction (002) peak at 24.4° and a significant (100) peak at 43.7° corresponding to the hexagonal graphitic carbon. The (100) peaks for the samples activated at 800 °C and 900 °C were found at 43.5° and 43.8°. The basal reflection (002) plane indicates the presence of few layer graphene nanosheets with hexagonal graphitic carbon and these results agree very well with the reported literature [42,43]. A broad peak indicates the amorphous nature of the material due to the presence of nitrogen atoms doped into the defective sites of the sheets [26]. The *d* spacing, i.e., the plane-to-plane distance between the graphene layers, is 3.6 Å when the aerogels are activated at 700 °C, 3.57 Å for 800 °C and 3.55 Å for 900 °C. These results show that when the material is activated at 900 °C, the interplanar distance is reduced; giving inference that the abundance of functional groups is reduced with increased activation temperature.

The crystallite size of the carbon aerogels was calculated using the Scherrer formula and was found to be 9.64, 8.17 and 8.51 nm for the materials activated at 700, 800 and 900 °C, respectively, suggesting better crystallinity, i.e., greater single crystal graphene domains for the former. These values are greater than those reported for traditional carbon aerogels prepared via the pyrolysis of sol–gel-derived resin precursors at 2500 °C (at ∼5 nm [44]), yet lower than those reported for aerogels produced by subjecting a graphene oxide-based 3D graphene to high-temperature annealing (at crystallite sizes of >40 nm, and as high as 150 nm [34]). It shows that the crystallite size decreases with respect to the activation temperature which is due to the presence of defects due to doping levels of nitrogen and this is consistent with the earlier reported work [45,46].

Raman spectra for the three samples were taken to elucidate the structural characteristics of carbon nanostructures produced in this study, with the representative spectra shown in Figure 4b. The shape of all spectra appears to be very similar to the Raman spectra of the previously reported traditional graphene-based aerogels carbonized at <1500 °C, with prominent, broad D and G bands and weak, poorly-defined D′ and G′ bands [34,44,47]. The spectra show three peaks: G, D and 2D peaks at 1590, 1350, 2761 cm^−1^, respectively. The G peak denotes the bond stretching of sp^2^ atom pairs, the D peak is related to the breathing mode of sp^2^ aromatic rings or defects and disorder carbon in graphene that is perpetuated by the presence of defects; and the 2D peak. The latter is an overtone of the D peak, however it is seen in the absence of defects. The crystal size which provides an indication of the extent of the disorder is given by the ratio I_D_/I_G_ of the D and G peaks, which for the samples investigated here gave a value of 0.8429 for 700 °C, 1.14 for 800 °C, 1.21 for 900 °C [48,49]. This suggests that the activation at 900 °C results in the most defective carbon, with 700 °C samples being the most graphitized and 800 °C samples possessing the properties that are somewhat in between the other sample types. The increase in the D band intensity was due to the doping of nitrogen, where all of the nitrogen atoms occupied the defective sites, causing the D band to increase [50]. The upshift from I_D_/I_G_ ratio clearly shows that there is more disorder when the materials are activated at 900 °C, providing further evidence that there is more nitrogen being incorporated into the material [48,51]. The 2D peak provides an indication of the graphene-like properties in the structure. Broad peaks of 2D band show that there is a presence of graphene in few layers, which proves the 3D nature of the networks [26,49]. Since the peak intensity of the 2D band for 800 °C is higher than that for 900 °C and 700 °C, there are less layers in the materials activated at 800 °C compared to that activated at 900 °C and 700 °C.

Figure 4c shows the representative XPS spectrum for the samples activated at 800 °C to probe the chemical state of the C and N present in the 3D nitrogen self-doped graphene networks. From this graph, it is evident that the survey scan shows three peaks located at 284.5, 402 and 531 eV that correspond to C, N and O 1s, respectively. The ratio of the integrated peak areas of C:N:O is 1.035:1:1.574. The deconvolution analysis of these peaks is described below.

Figure 4d shows the functional groups present in the 3D nitrogen self-doped graphene-based carbon aerogels, when activated at 700, 800 and 900 °C. The presence of the different surface functional groups present in the aerogel activated at 700 °C was confirmed by the FTIR analysis. The band around 3445 cm^−1^ arose from the stretching vibrations of the O−H bonds, suggesting a weak distribution of O−H bonds due to physical adsorption of moisture. The band at 2922 cm^−1^ shows the stretching vibrations of CH_2_ and the peak at 2380 cm^−1^ shows the stretching vibration of C−H. The band at 1592 cm^−1^ is related to the C=C vibrations due to the presence of a benzene group, whereas the band at 1396 cm^−1^ is related to the vibrations of C−O due to the oxygen becoming settled in the defective sites. The band at 1068 cm^−1^ was related to the vibrations of C−N, confirming the presence of pyrrolic N. When aerogels were activated at higher temperatures, namely at 900 and 800 °C, additional heat resulted in the elimination of OH groups and increased the intensity of peaks associated with un-oxidized graphitic domains. Thus, for the samples activated at 900 and 800 °C, sharp peaks around 3800−3500 cm^−1^ related to the stretching vibrations of O−H bonds could be observed, as shown in Figure 4e. The peak around 2354 cm^−1^ is attributed to the stretching vibrations of the C−H bond, whereas the peak at 1697 cm^−1^ shows the stretching vibrations of C=O bonds. There is a peak shift from 1592 cm^−1^ to 1527 cm^−1^ at 800 °C and 1516 cm^−1^ at 900 °C, suggesting the disappearance of C=C bonds and the appearance of C=N bonds, providing evidence that the pyridinic N is getting doped into the material. A small peak at 1052 cm^−1^ shows the stretching vibration of C−N due to the pyrrolic N [26]. The high content of pyridinic N and graphitic N is due to the enhanced interaction rate of nitrogen atoms with the active sites of carbon-vacant sites during the higher activation temperatures [52,53].

**Figure 5:**
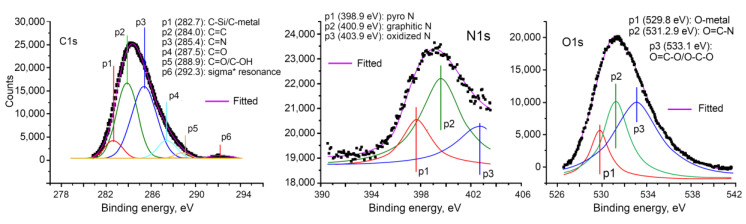
Deconvolution of the carbon, nitrogen and oxygen peaks of the general XPS spectra (Figure 4c) of the 3D nitrogen self-doped graphene-based carbon aerogel activated at 800 °C.

The deconvolution analysis of the C, N and O peaks is shown in Figure 5. The C 1s peak at 284.0 eV is due to C=C bond where sp^2^ carbon atoms exist in the conjugated honeycomb lattice [26,54]. Peaks at 287.5 and 288.9 eV suggest that carbon is bonded to oxygen as C=O and C=O/C–OH, respectively. The peak corresponding to C=N bonding is observed at 285.4 eV. It is well understood that the nitrogen doped into the graphene network experiences three bonding situations. Namely, it can be present as pyridinic N, pyrrolic N and graphitic nitrogen with corresponding peaks located at around 399.3, 400.5 and 401.8 eV respectively.

Data from the N 1s scan can be fitted with three peaks at 398.9, 400.9 and 403.9 eV, that correspond to pyrrolic, graphitic and oxidized nitrogen, respectively. The peak for pyridinic nitrogen (where N has a lone electron pair and is situated either at the edge of the graphitic lattice or near a vacancy, and bonded to two C) typically occurs at 398.3 ± 0.3 eV, however peaks falling within a broader range of 397.9–399.8 eV have also been reported as pyridinic nitrogen, possibly because the precise atomic configuration of these sites remain not fully understood [53]. For the nitrogen atom within a five-membered ring, the so-called pyrrolic nitrogen, the peaks were reported to occur around 400.1 ± 0.3 eV, however it should be noted that other signals, such as that of an N substitution in a Stone–Wales defect, and otherwise asymmetric local bonding could be responsible, with signals from amine, pyridone, nitroso and cyano groups possibly falling closely to this binding energy [53]. Thus, while informative, the deconvolution of XPS data alone is often not sufficient to categorically define the precise atomic structure of nitrogen-doped graphene aerogel. In our study, the XPS data is in broad agreement with the FTIR data, which suggests the presence of graphitic, pyrrolic and pyridinic nitrogen. Based on the combined results of the FTIR and XPS, it is likely that in the material activated at 800 °C, graphene nitrogen impurities are present in the substitutional (graphitic), pyrrolic and pyridinic configurations, where the latter could take the form of an edge pyridinic nitrogen, or as a single or triple N pyridinic vacancy. Thus, the synergistic pseudocapacitive interactions of the negatively charged pyrrolic N and pyridinic N groups, as well as the positive charge on the quaternary N contributes for the electron transfer through the graphene network, enhancing the conductivity of the graphene-based carbon aerogel [55].

### 3.4. Formation Mechanism of 3D Nitrogen Self-Doped Graphene

Gelatin dissolved in water is cross-linked with glutaraldehyde solution to form a gel. The gel formed is subjected to deep freeze and then lyophilized to obtain a 3D structured aerogel. This aerogel is carbonized at 800 °C, which decomposes the amine group to N and H. These N atoms released from the amine group replace some of the carbon atoms from the carbon lattice, thereby enabling the nitrogen self-doping to take place. Further KOH activation is done at high temperatures (700, 800 and 900 °C). During the KOH activation, H_2_O, K_2_CO_3_ and K_2_O are formed as a result of the reaction between KOH and the amorphous carbon; these are removed by the subsequent washing process. The etchant bestows the amorphous portion of carbon aerogels with pores. The high surface energy of amorphous carbon increases the probability of reactions with KOH than the reaction of crystalline graphene with KOH.

The KOH activation was performed at different temperatures so that active the surface area and pore distribution could be optimized. At 700 °C it is noted that due to insufficient temperature the amorphous carbon is not properly etched, whereas at 900 °C the vigorous etching driven by the high temperature is sufficient to leave behind a pure graphene framework. At 800 °C, it is possible to achieve a desirable fraction of amorphous carbon and nitrogen within the 3D graphene network.

### 3.5. Electrochemical Studies

**Figure 6:**
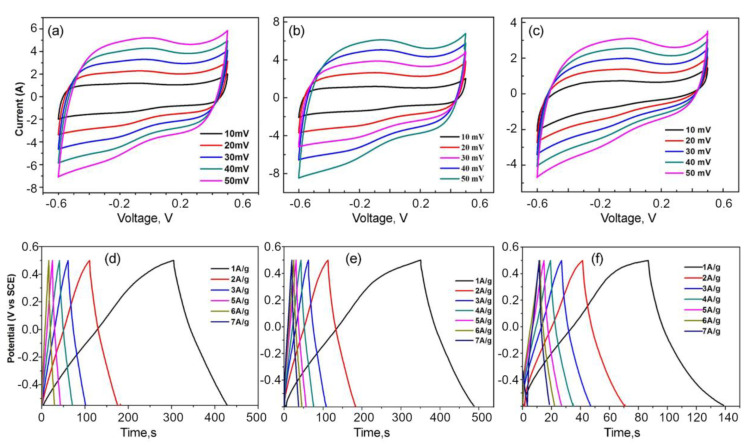
Cyclic voltammetry (CV) plots of the 3D nitrogen self-doped graphene-based carbon aerogel activated at (**a**) 700 °C, (**b**) 800 °C, (**c**) 900 °C. Galvanostatic charge/discharge curves of the 3D nitrogen self-doped graphene-based carbon aerogel activated at (**d**) 700 °C, (**e**) 800 °C, (**f**) 900 °C.

The electrochemical performance of the three electrode materials synthesized were first evaluated using cyclic voltammetry (CV) against SCE with a potential range of −0.6 V to 0.6 V at different scan rates in 1M KOH electrolyte, and the results are depicted in Figure 6a–c. The CV curves maintain a rectangular shape indicating the ideal capacitive behavior of the electric double layer capacitor (EDLC) carbonaceous material. Calculated using previously described formulae [56,57,58], the specific capacitance of carbonized samples activated at 700, 800 and 900 °C is 46 F× g^−1^, 112 F× g^−1^ and 51 F× g^−1^, respectively. Samples activated at 800 °C have the highest specific capacitance among all the samples at the same scan rate.

The accessible specific surface area and pore structure determines the capacitive behavior of the graphene-based nanostructures. The carbon sample activated at 700 °C is amorphous in nature and the surface area is also lower compared to the other two sample types, which is clearly observed from the BET results. Although the surface area is the highest for the sample heated at 900 °C, the specific capacitance of the sample heated at 800 °C is greater; this is due to the number of active pores present in the sample heated at 800 °C which is greater than the sample heated at 900 °C [26]. Samples heated at 800 °C show excellent supercapacitive behavior compared to the samples heated at 700 and 900 °C. This is due to the optimum distribution of the amorphous carbon and nitrogen in the 3D graphene. In addition to the EDLC behavior, the nitrogen species self-doped on the carbon surface contribute for excellent pseudocapacitance behavior by interacting with electrolyte ions. Secondly, the redox activity of oxygen also enhances the overall capacitance for supercapacitors. Thus, the synergistic effect of graphene and porous amorphous carbon network ensures good conductivity and enhances charge transport and storage in graphene-based aerogels activated at 800 °C.

The synthesized materials were further examined using charge/discharge measurement. The galvanostatic charge–discharge profiles for all three samples were conducted at various current densities, which are shown in Figure 6d,e. From the graph, the materials are inferred as good EDLC materials with high columbic efficiency, as the triangular shape of the graph has good symmetry with the nearly linear slope. The specific capacitance is calculated from the discharge curve using the Equation (2) as reported earlier, and the specific capacitance at a current density of 2 A× g^−1^ is found to be 232, 236, and 170 F× g^−1^ for the samples heat treated at 700 °C, 800 °C and 900 °C, respectively. From the above values, we come to a conclusion that the material activated at 800 °C has higher specific capacitance than the other two samples which is due to the high-surface area provided by an increased number of active pores and a combination of the amorphous and crystalline nature of the material.

**Figure 7:**
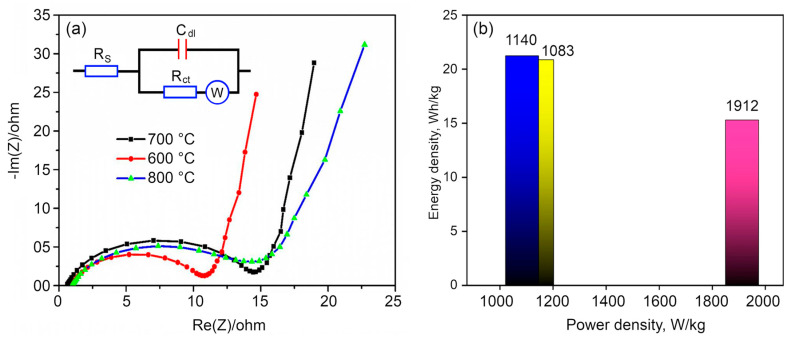
(**a**) Electrochemical impedance spectrum (EIS) measurements of the 3D nitrogen self-doped graphene-based carbon aerogel activated at 700 °C, 800 °C, and 900 °C. (**b**) The Ragone plot of the 3D graphene nitrogen-doped carbon aerogel activated at 700 °C (yellow bar), 800 °C (blue bar) and 900 °C (pink bar).

The electrochemical impedance spectrum (EIS) was obtained to gain a better understanding of electrode activity. The EIS study explains the ionic conductivity and resistivity of electrode materials. The value of ESR relates to the electrical conductivity of the electrode material. If the sample shows a lower ESR value, it corresponds to the highest electrical conductivity. To study the series resistance and charge transfer resistance of the electrodes, Nyquist plots of the sample activated at different temperatures are shown in Figure 7a. An ideal supercapacitor is characterized by a small series resistance and a charge transfer resistance. From the graph, it can be observed that a high-frequency semicircle followed by the sloped line at a low frequency which indicates the capacitive characteristic nature of the electrodes. This can be assigned to the Warburg impedance and diffusive resistance which mainly arises by an ion transport onto the electrode surface. The visible high frequency semicircle for all the samples can be attributed to the charge–transfer resistance in the electrode surface. The electrodes based on the samples activated at 700 and 900 °C showed the higher charge transport resistance when compared to the samples activated at 800 °C. This observation is in good agreement with the CV and charge–discharge analysis. The observed Nyquist plots were fitted with an equivalent circuit shown in the inset in Figure 7a. The estimated equivalent series resistance (ESR) values were found to be 14.3 Ω, 9.5 Ω and 14 Ω for 700 °C, 800 °C and 900 °C, respectively. The low ESR value of the samples activated at 800 °C is indicative of the high electrical conductivity of the electrode due to the highly porous structure with uniform pores and its distribution when compared to the other two samples. At the same time, the quartanery N or graphitic Nitrogen configuration with no free electron lone pairs on the N atoms is considered to only enhance the electron conductivity in graphene populating the conduction band [59]. The samples carbonized at 700 °C, shows a concealing or masking of the graphene layers by the large amount of amorphous carbon. After the KOH activation, some of the amorphous carbon is etched away, leaving behind some part of the amorphous carbon and the highly graphitized graphene layers in the 3D nitrogen self-doped graphene. Samples activated at 700 and 900 °C showed a lower conductivity when compared with those activated at 800 °C, which is characterized by a smallest charge transfer resistance of about 9.5 Ω. Hence, from cyclic voltammetry, galvanostatic charge/discharge and EIS study, it is seen that the sample heated at 800 °C showed a better supercapacitive behavior than the samples heated at 700 and 900 °C.

Furthermore, the correlation between the power and energy densities of the synthesized materials was investigated using the Ragone plot, which is shown in Figure 7b. The power and energy density values confirm the EDLC behavior of the prepared materials. The energy densities of the samples activated at 700, 800 and 900 °C samples are 20.88, 21.24 and 15.3 Wh/kg, respectively. The samples activated at 700 and 800 °C have almost similar power and energy densities, however, the samples activated at 900 °C have the highest power density with lower energy density compared to the other materials. This infers that the property of the aerogels activated at 900 °C was drastically changed by increasing the temperature of activation.

**Figure 8:**
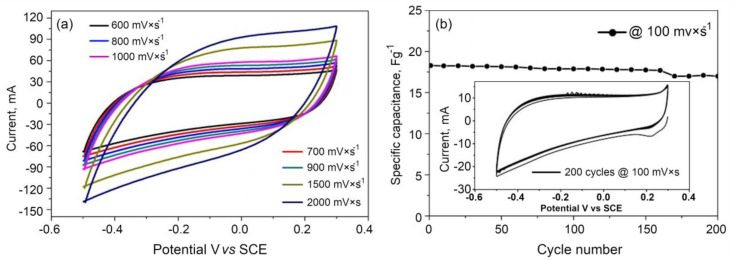
(**a**) CV analysis of the aerogel activated at 800 °C at a higher scan rate, and (**b**) the stability of the 3D carbon aerogel activated at 800 °C.

Figure 8 demonstrates the stability of the aerogel material activated at 800 °C when scanned at 100 mVs^−1^. The results show a negligible capacitance decay over the test cycles, with almost 98% of the capacitance being retained. This indicates the excellent cyclic stability of the thus fabricated aerogels that have been activated at 800 °C. The electrode material shows excellent stability at higher voltages. The material seems to be the high rate performance material as it can be swept through very high scan rates from 600 to 2000 mV/s, while maintaining a typical rectangular shape with only a slight deviation. As the material is shown to support up to 2000 mV/s, it is likely to be suitable for a high-performance supercapacitor electrode with a high-rate capability and cyclability, as well as for other electrode applications, such as in graphene-organic memory devices [60].

## 4. Conclusions

In this work, novel 3D nitrogen self-doped graphene materials were prepared from gelatin as the source material. Gelatin was cross-linked with glutaraldehyde to form a gel, then the gel was deep frozen and lyophilized to form an aerogel which was subsequently heat treated, and then exposed to temperatures of 700−900 °C and chemically activated by KOH to increase the porosity and surface area. Samples activated at 800 °C had the highest capacitance 236 F× g^−1^ at 2 A× g^−1^ among all the samples because of the large specific surface areas, as well as a high mesoporosity and superior electronic conductivity compared to other samples. These samples also showed the highest energy density amongst the three sample types, with the samples activated at 700, 800 and 900 °C showing energy densities of 20.88, 21.24 and 15.3 Wh/kg, respectively. These results suggest that 3D nitrogen self-doped graphene aerogels activated at 800 °C have the potential to be used as high-performance electrode materials in energy storage applications.

## Figures and Tables

**Table 1:** Surface area and pore characteristics of graphene-based carbon aerogels activated at 700, 800, and 900 °C.

Sample Activation Temperature, °C	Surface Area m^3^/g	Pore Volume cm^3^/g	Pore Diameter, nm
700	230	0.24	4.55
800	1539	0.88	19.30
900	1420	0.78	17.00
